# Primary open anterior shoulder stabilization: a long-term, retrospective cohort study on the impact of subscapularis muscle alterations on recurrence

**DOI:** 10.1186/1471-2474-15-45

**Published:** 2014-02-20

**Authors:** Axel Gamulin, Romain Dayer, Anne Lübbeke, Hermes Miozzari, Pierre Hoffmeyer

**Affiliations:** 1 Division of Orthopedic and Trauma Surgery, Department of Surgery, University Hospitals of Geneva, 4 Rue Gabrielle-Perret-Gentil, CH-1211 Geneva 14, Switzerland

**Keywords:** Post-traumatic anterior shoulder instability, Primary shoulder stabilization, Open labral repair, Open capsulorrhaphy, Recurrence, Subscapularis muscle, Histopathologic lesions

## Abstract

**Background:**

Post-traumatic anterior shoulder instability patients may present histopathologic lesions within the subscapularis muscle compatible with a scarring process associated with disuse atrophy. We hypothesized that such lesions identified on intraoperative biopsy at the time of primary anterior shoulder stabilization would predict a higher risk of postoperative instability recurrence.

**Methods:**

Of 52 eligible patients (52 shoulders) who had undergone subscapularis muscle biopsy during primary anterior open labral repair and capsulorrhaphy, 35 (mean age at surgery, 27.2 years; male sex, 71.4%) were retrospectively evaluated (mean follow-up, 12.9 years; range, 10.9-14.5 years). Primary outcome was shoulder instability recurrence. Secondary outcomes included shoulder range of motion, functional scores, and radiological analysis of glenohumeral joint degenerative changes.

**Results:**

Overall five patients (14.3%) presented shoulder instability recurrence. Twelve patients with histopathologic lesions had significantly more instability recurrence than 23 without histopathologic changes (33.3% vs. 4.3%; risk difference, 29% [95% CI 1; 57]; p = 0.038). Patients without histopathologic changes had significantly reduced external rotation with arm at side (ER1; -11.9°; p = 0.001) and with shoulder abducted to 90° (ER2; -14.9°; p = 0.001) on the operated side when compared to the contralateral side. Patients with histopathologic lesions had only ER2 significantly reduced (-8.9°; p = 0.031). There was no substantial difference regarding functional and quantitative radiological scores between both patients’ groups.

**Conclusions:**

Histopathologic changes within the subscapularis muscle at the time of primary open labral repair and capsulorrhaphy were associated with an increased risk of shoulder instability recurrence. Further investigations are needed to assess the impact of dedicated postoperative rehabilitation programs for patients presenting these lesions. Their recognition on preoperative magnetic resonance imaging should also be investigated; non-anatomical repairs could be an option in these cases.

## Background

Open labral repair associated with capsulorrhaphy remains the gold standard for post-traumatic anterior shoulder instability when conservative treatment is ineffective [1,2]. The reported risk of postoperative shoulder instability recurrence with a follow-up >10 years ranges from 4.5% to 16.1% [3-6]. Initial arthroscopic techniques had unacceptably high long-term recurrence risks, exceeding 30% in two case series with a mean follow-up >10 years [7,8]. Recent arthroscopic advances showed short-term results similar to open techniques, but longer follow-up are needed [1,9,10].

A histomorphometric study demonstrated that approximately 40% of patients with post-traumatic anterior shoulder instability presented with histopathologic lesions in the substance of the subscapularis muscle at the time of surgery [11]. These lesions were characterized by type 1 fiber predominance, interstitial fibrosis, and focal atrophy compatible with a scarring process associated with disuse atrophy. As the subscapularis muscle biomechanically acts as one of the major anterior stabilizers of the shoulder joint, there may be a subset of patients more prone to instability recurrence after surgical stabilization [11-14].

We hypothesized that such lesions within the subscapularis muscle identified on intraoperative biopsy would predict a higher risk of postoperative recurrence of shoulder instability. This study retrospectively compared the long-term functional and radiological outcomes after open labral repair and capsulorrhaphy for post-traumatic anterior shoulder instability in patients with and without histopathologic lesions in the subscapularis muscle.

## Methods

### Ethics statement

Before the initiation of the study, approval was obtained from the institutional research ethics board (namely, *Comité départemental d'éthique de neurochirurgie, anesthésie et chirurgie,* a subdivision of our medical center’s *Commission centrale d'éthique de la recherche sur l'être humain*). All participants provided written informed consent.

### Study participants

We conducted a retrospective cohort study in a 1,900-bed urban academic medical center providing primary and tertiary care for 500,000 inhabitants. Patient inclusion criteria were: 1) a primary open labral repair associated with capsulorrhaphy for post-traumatic anterior shoulder instability; 2) a subscapularis muscle biopsy at the time of surgery; 3) >16 years old at the time of surgery; and 4) provided written informed consent. Patients operated for revision of a failed stabilization or multidirectional or voluntary shoulder instability were excluded. Fifty-two consecutive patients (52 shoulders) operated between November 1994 and February 1998 and reported in a previous investigation were eligible for inclusion [11]. Before first dislocation, all patients practiced recreational sports activities. There were no high-level athletes. Seventeen patients (32.7%) were not available for follow-up: nine could not be located despite intensive tracking efforts; six refused to participate because of a heavy workload (four), poor general health status (one), or conflict with our institution (one); one had chronic psychiatric disorders preventing her participation in the study; one had died from reasons unrelated to the surgery. Eventually, 35 patients (67.3%) were included in the final analysis.

### Operative technique

The senior author performed all surgeries. The procedure was a modified Bankart repair (without coracoid osteotomy) using absorbable sutures to repair the labrum [15,16]; in each case, a laterally-based T-shaped capsulorrhaphy was associated [17]. Intraoperativelly, one biopsy specimen was harvested from the lower third of the subscapularis muscle, 1 cm medial to the tendon. This site was selected because the subscapularis muscle is difficult to biopsy at any other location during surgery. The shoulder was immobilized for the first six postoperative weeks, with gentle pendular movements beginning on the third week, followed by progressive strengthening and range of motion (ROM) exercises. Return to work was allowed depending on the type of activity and hand dominance. Heavy labor was not allowed before three months following surgery. Sport activities were allowed from the third month, with overhead and contact sports delayed until six months after surgery. Of note, all patients were practicing sport at a recreational level.

### Exposure

Subscapularis muscle biopsy analysis has been described in a previous report [11]. It included connective tissue assessment and muscle fiber morphologic study, typing (type 1 fibers, slow and aerobic, and type 2 fibers, rapid and anaerobic) and counting. Results were recorded from patients’ charts and categorized into normal and abnormal findings. Abnormal histology was defined as histopathologic changes compatible with a scarring process (interstitial fibrosis) associated with disuse atrophy (type 1 fiber predominance, focal atrophy), and normal histology as an absence of these anomalies [11].

### Outcomes

The primary outcome measure was recurrence of postoperative shoulder instability: it was defined as postoperative dislocation or painful subluxation with shoulder instability demonstrated by physical examination. Past history was obtained relative to postoperative episodes of dislocation or subluxation. Physical examination assessed stability (sulcus sign, load and shift test, anterior and posterior drawer tests) of both operated and contralateral shoulders [17-19].

Secondary outcomes were: 1) active ROM; 2) functional scores; and 3) quantitative radiological analysis of degenerative changes. Active ROM (flexion, extension, abduction, lateral elevation, internal rotation, and external rotation with arm held at side [ER1] and with shoulder abducted to 90° [ER2]) of both shoulders were measured with a goniometer at follow-up. Preoperative ROM were recorded from patients' charts. ROM values of contralateral pathological shoulders (five non-operated unstable shoulders and three surgically stabilized shoulders) were excluded from the data analysis.

Peak strength with the arm abducted to 90°, the elbow fully extended, and the forearm maximally pronated, was measured on both sides using a fixed-spring balance (Macro 35 kg, Pesola AG, Baar, Switzerland) to obtain a strength value for determination of the Constant-Murley shoulder score [20-22]. The Rowe shoulder score, the American Shoulder and Elbow Surgeons (ASES) shoulder assessment, and the Single Assessment Numeric Evaluation (SANE) rating were also determined [23-25]. The Constant-Murley shoulder score and the ASES shoulder assessment were measured for healthy contralateral shoulders. Preoperative ASES shoulder assessments were unavailable, since this score was not used at our institution at the time when surgeries were performed. The Constant-Murley shoulder score is not validated for patients undergoing surgery for shoulder instability [20]. We used this score at follow-up because it was already obtained preoperatively and it is reported in other long-term follow-up studies on open anterior shoulder stabilization, thus allowing for comparison [3,26,27]. The Rowe shoulder score, the ASES shoulder assessment and the SANE rating have been validated for the evaluation of patients after shoulder stabilization [25,28,29].

Preoperative and follow-up anteroposterior plain radiographs of the affected shoulder were reviewed blindly by three of the authors for the presence of degenerative changes. These changes were quantitatively graded using the Samilson and Prieto classification, which demonstrated a high level of agreement in the evaluation of glenohumeral osteoarthritis after open anterior shoulder stabilization according to interobserver reproducibility studies [30,31]. Degenerative changes were graded as mild if inferior glenoid or humeral osteophytes less than 3 mm high were observed; moderate if osteophytes were between 3 mm and 7 mm high or if there was slight irregularity of the glenohumeral joint; and severe if osteophytes were more than 7 mm high or if there was sclerosis and narrowing of the glenohumeral joint.

### Covariates

Age at first dislocation, surgery and follow-up, sex, side of injury, hand dominance, type of injury (major [motor vehicle accident, fall, sports injury] or minor trauma [forceful abduction with external rotation]), and preoperative Constant-Murley shoulder score, body mass index, and American Society of Anesthesiologists grade were recorded from patients’ charts. The number of preoperative dislocations was obtained from patient’s charts, and categorized into four groups: 1) one single dislocation; 2) 2-5 dislocations; 3) 6-10 dislocations; 4) >10 dislocations.

### Statistical analysis

To compare covariates between exposure groups, p values were obtained using the Mann-Whitney U test for continuous variables and Fisher’s exact and Pearson’s chi-squared tests for categorical variables. Risk difference and the 95% confidence interval (95% CI) were calculated to assess the effect of histopathologic anomalies on recurrence. Fisher’s exact test was used to obtain a p-value. Following comparison of active ROM of the operated side with the contralateral side, mean differences were calculated and p-values were obtained using the Wilcoxon signed-rank test. To compare outcome scores between exposure groups, mean differences and their 95% CIs were calculated and p-values were obtained using the Mann-Whitney U test. The IBM® SPSS® Statistics version 19.0.0 software (IBM SPSS, Chicago, IL) was used for statistical analysis. A value of p < 0.05 was considered statistically significant.

## Results

Thirty-five patients (67.3%) were available for evaluation at a mean follow-up of 12.9 years (range, 10.9-14.5 years). Thirty-four had a complete history and clinical evaluation. Among these, one refused to have radiographs. Another patient only had a phone interview and filled out a questionnaire, which provided data on history and clinical scores (except for the Constant-Murley shoulder score which requires measurement of abduction strength). Because of no history of postoperative shoulder dislocation or suspected painful subluxation, this patient was allocated to the non-recurrent group.

Twelve patients (34.3%) demonstrated histopathologic lesions on the subscapularis muscle biopsy. The remaining 23 (65.7%) presented normal subscapularis muscle histology. Both patients’ groups had similar demographic and injury characteristics, except for sex ratio and preoperative Constant-Murley shoulder score (Table 1).

**Table 1:** Demographics, injury characteristics, and preoperative Constant-Murley shoulder score of the study patients

		**Study cohort N = 35**	**Abnormal histology 12 patients (34.3%)**	**Normal histology 23 patients (65.7%)**	P Value*
Age (y) at	First dislocation	22.3 (13-53)	20.9 (13-37)	23.0 (13-53)	0.555
	Surgery	27.2 (16.8-54.9)	27.4 (16.8-43.2)	27.1 (17.3-54.9)	0.404
	Follow-up	40.1 (30.1-67.9)	40.5 (30.5-56.5)	39.9 (30.1-67.9)	0.404
Delay (y)	First dislocation–surgery	5.0 (1.1-28.5)	6.5 (1.3-28.5)	4.2 (1.1-15.2)	0.555
	Surgery-follow-up	12.9 (10.9-14.5)	13.1 (12.0-13.9)	12.8 (10.9-14.5)	0.404
Male sex		25 (71.4%)	6 (50.0%)	19 (82.6%)	0.059
Right side injured		14 (40.0%)	4 (33.3%)	10 (43.5%)	0.721
Dominant side injured		13 (37.1%)	5 (41.7%)	8 (34.8%)	0.726
Major trauma		34 (97.1%)	12 (100.0%)	22 (95.7%)	1.000
Number of preoperative dislocations					0.768
	1	1 (2.9%)	0	1 (4.3%)	
	2-5	12 (34.3%)	4 (33.3%)	8 (34.8%)	
	6-10	4 (11.4%)	2 (16.7%)	2 (8.7%)	
	>10	18 (51.4%)	6 (50.0%)	12 (52.2%)	
Preoperative BMI (kg/m^2^)		23.5 (16.8-30)	23.1 (16.8-28.1)	23.7 (19.5-30.0)	0.903
Preoperative ASA grade 1-2		35 (100.0%)	12 (100.0%)	23 (100.0%)	1.000
Preoperative constant		80.9 (52-100)	78.0 (53-97)	82.3 (52-100)	0.044

*P values were obtained by using the Mann-Whitney U test for continuous variables and Fisher’s exact and Pearson’s chi-squared tests for categorical variables.

Values are expressed as mean (range) for ages, delays, BMI, and Constant-Murley shoulder score, and N (%) for other variables.

*Abnormal histology* is defined as histopathologic changes within the substance of the subscapularis muscle compatible with a scarring process associated with disuse atrophy. *Normal histology* is defined as the absence of the above-mentioned anomalies. *Preoperative BMI*: preoperative Body Mass Index. *Preoperative ASA grade*: preoperative American Society of Anesthesiologists grade. *Preoperative Constant*: preoperative Constant-Murley shoulder score.

Five patients (14.3%) overall experienced recurrent shoulder instability, with at least one episode of shoulder dislocation. None demonstrated painful subluxations as the only sign of recurrent shoulder instability. Four of these five patients were among the 12 with subscapularis muscle anomalies corresponding to 33.3%, and the other one was among the 23 without histopathologic lesions corresponding to 4.3% (risk difference, 29% [95% CI 1; 57]; p = 0.038). The greater incidence of recurrence in the presence of histopathologic lesions as compared to no lesions was seen both in men (33.3% vs. 5.3%, respectively) and in women (33.3% vs. 0%, respectively).

At follow-up, active ROM on the operated side was similar to the contralateral side for the entire cohort, except for external rotation, with a mean difference of 10.0° for ER1 and 12.7° for ER2 (p < 0.001 for both values) (Table 2). Among patients without histopathologic lesions, ER1 and ER2 were significantly lower on the operated side when compared to the contralateral side (-11.9° ER1, p = 0.001; -14.9° ER2, p = 0.001) (Table 3). Among patients with histopathologic lesions, ER2 alone was significantly lower on the operated side (-8.9°, p = 0.031).

**Table 2:** Comparison of active shoulder range of motion at follow-up between operated side and contralateral side

	Operated side (N = 34)*	Contralateral side (N = 26)*	**Mean difference**	**P Value†**
Flexion (°)	178.7 (155-180)	180.4 (180-190)	-1.7	0.317
Extension (°)	59.7 (25-90)	61.7 (35-90)	-2.0	0.066
Abduction (°)	169.3 (90-180)	174 (130-180)	-4.7	0.197
Lat. elevation (°)	177.6 (160-180)	178.5 (160-190)	-0.9	0.414
IR‡	5.6 (2-10)	4.8 (1-8)	0.8	0.437
ER1 (°)	60.6 (35-80)	70.6 (40-90)	-10.0	<0.001
ER2 (°)	78.5 (50-100)	91.2 (70-110)	-12.7	<0.001

*Values are expressed as mean (range).

†P values were obtained by using the Wilcoxon signed-rank test.

‡Number of the thoracic vertebra reached by the thumb: the first thoracic vertebra is numbered 1, proceeding to the twelfth thoracic vertebra, which is numbered 12.

*Contralateral side:* values from contralateral pathological shoulders (five non-operated unstable shoulders and three surgically stabilized shoulders) were excluded for the data analysis to obtain a healthy shoulder group for comparison. *Lat. elevation*: lateral elevation. *IR*: internal rotation. *ER1*: external rotation with arm held at side. *ER2*: external rotation with shoulder abducted to 90°.

**Table 3:** Active shoulder external rotation at follow-up

	**Operated side, abnormal histology (N = 11)**			**Operated side, normal histology (N = 23)**			**Contralateral side (N = 26)**
	**Mean (range)**	Mean diff.*	**P Value†**	**Mean (range)**	Mean diff.*	**P Value†**	**Mean (range)**
ER1 (°)	64.5 (35-80)	-6.1	0.062	58.7 (35-80)	-11.9	0.001	70.6 (40-90)
ER2 (°)	82.3 (70-100)	-8.9	0.031	76.7 (50-100)	-14.5	0.001	91.2 (70-110)

*Mean difference between active external rotation on operated side and active external rotation on contralateral side.

†P values were obtained by using the Wilcoxon signed-rank test.

*Abnormal histology* is defined as histopathologic changes within the substance of the subscapularis muscle compatible with a scarring process associated with disuse atrophy. *Normal histology* is defined as the absence of the above-mentioned anomalies. *Contralateral side:* values from contralateral pathological shoulders (five non-operated unstable shoulders and three surgically stabilized shoulders) were excluded for the data analysis to obtain a homogenous native shoulder group for comparison. *Mean Diff.*: mean difference. *ER1*, external rotation with arm held at side. *ER2*: external rotation with shoulder abducted to 90°.

The mean Constant-Murley shoulder score of the affected side increased for the entire cohort from a preoperative value of 80.9 to 92.8 at follow-up (gain, 11.9, 95% CI 8.5; 13.7). Mean contralateral side values were 96 before surgery and 96.2 at follow-up (gain, 0.2). At final evaluation of the entire study group, the mean Rowe shoulder score was 87.4, the mean ASES shoulder assessment 93.1 (contralateral side, 97.0), and the mean SANE rating 88.7. There was no statistically significant difference in these scores between patients with and without subscapularis muscle histopathologic anomalies (Table 4).

**Table 4:** Functional scores at follow-up

	Abnormal histology (N = 12*)†	**Normal histology (N = 23)†**	**Mean difference‡ (95% CI)**	P Value**
Constant	91.9 (80-100)	93.2 (58-100)	-1.3 (-7.6; 5.1)	0.482
Gain	11.6	10.8	-0.8 (-4.6; 6.2)	0.868
Rowe	78.8 (40-100)	92.0 (45-100)	-13.2 (-30.2; 3.8)	0.271
ASES	93.2 (73-100)	93.1 (43-100)	0.1 (-8.7; 8.7)	0.311
SANE	90.3 (80-100)	87.9 (50-100)	2.4 (-6.5; 11.3)	0.873

*Except for Constant-Murley shoulder score and related gain, N = 11; see Methods section for explanation.

†Values are expressed as mean (range).

‡Mean difference between scores of patients with abnormal and normal histology.

**P values were obtained by using the Mann-Whitney U test.

*Abnormal histology* is defined as histopathologic changes within the substance of the subscapularis muscle compatible with a scarring process associated with disuse atrophy. *Normal histology* is defined as the absence of the above-mentioned anomalies. *95% CI*: 95% confidence interval. *Constant*: Constant-Murley shoulder score. *Gain*, gain between preoperative and postoperative Constant-Murley shoulder scores. *Rowe*, Rowe shoulder score. *ASES*, American shoulder and elbow surgeons shoulder assessment. *SANE,* single assessment numeric evaluation rating.

Degenerative changes were absent on preoperative radiographs in all cases. Quantitative evaluation of degenerative changes on follow-up radiographs is shown in Table 5. There was no statistically significant difference between patients with and without histopathologic changes. There was no correlation between severity of degenerative changes and number of dislocations before surgical stabilization.

**Table 5:** Quantitative radiological evaluation of glenohumeral joint degenerative changes at follow-up

	Study cohort N = 33*	Abnormal histology N = 10*	**Normal histology N = 23**	**P Value†**
Degenerative changes				0.351
No	18 (54.5%)	5 (50.0%)	13 (56.5%)	
Mild	12 (36.4%)	3 (30.0%)	9 (39.1%)	
Moderate	3 (9.1%)	2 (20.0%)	1 (4.3%)	
Severe	0	0	0	

*Two patients did not have follow-up radiographs.

†P value was obtained by using the Pearson’s chi-squared test.

Values are expressed as N (%).

*Abnormal histology* is defined as histopathologic changes within the substance of the subscapularis muscle compatible with a scarring process associated with disuse atrophy. *Normal histology* is defined as the absence of the above-mentioned anomalies. *Degenerative changes*: see Methods section for a full description of the grades of the Samilson and Prieto classification of glenohumeral joint degenerative changes.

## Discussion

This study reports that 12.9 years after primary open labral repair and capsulorrhaphy for post-traumatic anterior shoulder instability, patients presenting with specific histopathologic changes within the subscapularis muscle at the time of surgery had a significantly increased incidence of recurrence compared to patients with normal histology (33.3% vs. 4.3%, respectively). Except for external rotation, the presence of these tissue alterations did not affect other clinical and radiographic outcomes. Indeed, ER1 was significantly decreased on the operated compared to the contralateral side in patients with normal histology (-11.9°), but not in patients with abnormal histology (-6.1°).

This study sample closely relates to the population undergoing shoulder stabilization for post-traumatic anterior shoulder instability, with patients’ demographic characteristics (sex ratio, mean age) matching those recently reported in a dedicated multicenter register [32].

The overall incidence of recurrence in our cohort (14.3%) is within the range reported by other authors with a follow-up >10 years after the same procedure and including painful subluxation in the definition of recurrence (4.5% to 16.1%) [3-6]. An association was found between histopathologic anomalies of the subscapularis muscle (type 1 fiber predominance, interstitial fibrosis and focal atrophy) and recurrence of shoulder instability. To our knowledge, this association has never been reported yet. A possible explanation could be that the anterior stabilizing function of the subscapularis muscle is adversely affected by a scarring process associated with disuse atrophy within its substance. Decreased muscular tone resulting in failure of dynamic shoulder stabilization and some degree of laxity could lead to an increased risk of recurrent shoulder instability [11]. These histopathologic anomalies might correspond to the macroscopic lesions of the same muscle (muscular tears seen intraoperatively) described by Rowe et al. in 7% of 158 unstable shoulders [23]. When compared to the 40% prevalence of microscopic lesions observed in our previous report, this lower prevalence of macroscopic tears leads us to hypothesize that all these lesions may belong to a unique pattern of progressive muscle injury, with macroscopic tears representing a more advanced stage [11]. These lesions could be related to the initial traumatic dislocation and recurrent episodes, to the difficulty in reduction, and to the healing process within the injured muscle. However, the presence of histopathologic changes in the subscapularis muscle was not associated with an increased number of dislocations before surgical stabilization. This suggests that the initial traumatic dislocation and difficulty in reduction may be more important than the number of dislocation episodes before stabilization in the development of these histopathologic changes.

Active ROM at follow-up revealed a significant decrease of mean ER1 (-10.0°) and ER2 (-12.7°) of the operated shoulder for the whole study cohort. Other studies with a follow-up >10 years after the same procedure have shown similar results (reported decreases ranging from 6° to 33.6° for ER1 and from 8.7° to 24.4° for ER2) [3-6,26,33-35]. The presence of histopathologic lesions affected differently the decrease of ER1 and ER2 with no significant decrease of ER1 on the operated side among patients with histopathologic changes. Open labral repair and capsulorrhaphy is a procedure restricting external rotation due to tightening of anterior soft tissues [6,23,36]. The absence of a significant postoperative decrease of ER1 in patients with subscapularis muscle histopathologic lesions could be linked to reduced passive resistance and attenuated dynamic shoulder stabilization activity of the pathologic muscle.

Functional follow-up scores confirmed the good long-term results already reported in the literature after the same procedure [3-6,26,27,34]. Although patients with subscapularis muscle histopathologic anomalies had more recurrence in this study, their functional scores at follow-up were not significantly different when compared to patients with normal muscle histology. However, they presented with a trend towards a lower Rowe shoulder score. This is mainly explained by the fact that one-third presented recurrence of shoulder instability and half of this score assesses stability.

At follow-up, degenerative changes were absent in nearly half of all patients, mild in one third, moderate in one tenth, and severe in none. The severity of these changes was not correlated to the number of preoperative dislocation episodes. Results from other studies with follow-up >10 years suggest that primary open labral repair and capsulorrhaphy for post-traumatic anterior shoulder instability are associated with progressive shoulder osteoarthritis developing over the years [3-6,26,27,34]. This process could possibly be caused by the physiologic interference of the surgical repair. Osteoarthritic changes have been associated with loss of external rotation and overtightening of anterior soft tissues around the glenohumeral joint following stabilization [5,6,36,37]. The present study showed that patients with subscapularis muscle histopathologic changes had less loss of external rotation than patients without these anomalies. Consequently, we would have expected them to have less degenerative changes. However, there was no statistically significant difference regarding degenerative changes between both patients' groups. This may be because of a too small sample size with insufficient statistical power to obtain significance, a possible issue with the validity of the Samilson and Prieto classification not currently documented, or because degenerative changes are primarily due to the traumatism of the initial dislocation [38,39].

This study has several limitations: 1) the bone loss on the glenoid rim or the humeral head (an important factor for recurrence after shoulder stabilization), which could not be evaluated as a confounding factor because it was neither consistently recorded on the operative notes nor clearly identifiable on available preoperative radiographs. Of note, we were able to review anteroposterior plain radiograph in neutral rotation for each patient, which was the routine in-hospital preoperative workout in our institution at the time surgeries were performed; however, additional views (anteroposterior with internal and external rotation, axial, or CT-scan in some instances), which were performed before hospitalization as part of a complete work-out, were no more available for a large number of patients, because they had been lost by the patients, not kept by the archive department of our institution, or of a too bad quality to allow for an accurate evaluation of the bone stock. 2) The relatively high rate of patients lost to follow-up (32.7%), which may be explained by their young age (mean age at surgery, 27.2 years) and the long delay since surgery (mean follow-up, 12.9 years); 3) the biopsy specimen harvested in one limited area, which may not imply that a significant portion of the subscapularis muscle presents with the same histopathologic features; 4) the number of preoperative shoulder dislocations, which may have been imprecisely recorded (relying on history taking); 5) the retrospective assessment of shoulder dislocation recurrence; and 6) the small number of events with respect to our primary outcome, leading to large confidence intervals.

## Conclusions

This is the first study to highlight an association between subscapularis muscle histopathologic anomalies and recurrence of shoulder instability. The presence of subscapularis muscle histopathologic changes predating surgery is a useful predictor of recurrence of shoulder instability, with a risk difference of 29% when compared to patients with normal subscapularis muscle biopsies. However, our study is unable to determine the importance of these changes compared with glenohumeral bone loss, which also influences recurrence. Further investigations are needed to assess the impact of specific postoperative rehabilitation programs for patients with subscapularis muscle histopathologic lesions in order to potentially improve the outcome of surgery. The association of these lesions with possible specific preoperative magnetic resonance imaging features (to date not described) should be investigated also in a correlation study. Non-anatomical repairs could be an interesting option in this subgroup of patients with the hope to further reduce instability recurrence, but still needs to be investigated.

## Competing interests

The authors declare that they have no competing interests.

## Author’s contributions

AG carried out the conception and design of the study, contributed to the acquisition and interpretation of data and drafted and critically revised the manuscript. RD participated in the conception and design of the study, contributed to the acquisition and interpretation of data and drafted and critically revised the manuscript. AL participated in the conception and design of the study, performed the statistical analysis, helped in drafting the manuscript and critically revised it. HM contributed to the acquisition and interpretation of data and critically revised the manuscript. PM participated in the conception and design of the study and critically revised the manuscript. All authors read and approved the final manuscript.

## Pre-publication history

The pre-publication history for this paper can be accessed here:

http://www.biomedcentral.com/1471-2474/15/45/prepub
